# Identification of key genes controlling anthocyanin biosynthesis in the fruits of a bud variety of Tarocco blood-orange

**DOI:** 10.1186/s12870-025-06212-7

**Published:** 2025-02-20

**Authors:** Peian Zhang, Quan Zhao, Yang Song, Huanchun Jin, Yingyao Liu, Dan Hu, Dongfeng Liu

**Affiliations:** 1 Zhejiang Institute of Subtropical Crops, Wenzhou, China; 2 Wenzhou Agricultural Technology Extension and Service Center, Wenzhou, China

**Keywords:** Tarocco blood orange, Bud variety, Transcriptome, Anthocyanin, Fruit quality trait

## Abstract

Tarocco is a prevalent blood-orange variety in China, has a bud variant identified in Wenzhou City. To characterize the quality traits and molecular mechanisms underlying inhibition of anthocyanin synthesis in this variety, we collected fruits of Tarocco (WT) and the bud variant Ouya (MT) at nine developmental stages. Their anthocyanin, soluble sugar, and organic acid profiles were examined, and transcriptomes and metabolites were analyzed at three developmental stages. The results revealed that MT is a new blood orange variety with weak anthocyanins and a better sugar-acid ratio than the WT. The content of anthocyanin in MT fruits were significantly lower than those in WT fruits, especially cyanidin-like anthocyanins, while the flavone contents exhibited no remarkable variation. A total of 64 differentially expressed genes (DEGs), including five transcription factors (TFs), five methylation-related genes, and one flavonoid biosynthesis gene, were identified between WT and MT at three fruit developmental stages. The potential regulatory networks of these TFs were further constructed using weighted gene co-expression network analysis.Furthermore, in MT fruit treated with the 5-azacytidine, we observed hypomethylation of anthocyanins accumulated in the pulp and the promoters and genebodies of some anthocyanin synthesis-related genes. These results provide new insights into the influence of DNA methylation on anthocyanin accumulation in MT and also provide support for the promotion of MT as a new variety.

**Supplementary Information:**

The online version contains supplementary material available at 10.1186/s12870-025-06212-7.

## Introduction

The synthesis of anthocyanins, recognized as a critical determinant that exerts a profound impact on both the aesthetic and nutritional qualities of fruits, has long occupied a central position in the academic exploration of fruit quality [1]. Anthocyanins not only serve as an important basis for fruit color formation but also possess a range of valuable health-related benefits, such as enhancing visual acuity, preventing cardiovascular disorders, and reducing blood pressure [2]. Numerous studies have shown that the anthocyanin contents and color types of fruits are primarily determined by structural genes involved in flavonoid biosynthesis, including*PAL*,*C4H*,*4CL*,*CHS*,*CHI*,*F3H*,*ANS*,*DFR*,*UFGT*, etc [3,4]., together with transcription factors (TFs), in conjunction withthe activity of TFs such as*MYBs*,*bHLHs*,*WDRs*, and*BBXs*[5,6]. In addition, external environmental factors (including light, temperature, hormones, and fungi), as well as epigenetic modifications (e.g., DNA methylation), have been established to influence the levels of anthocyanins accumulated in fruits [7–10]. For example, DNA methylation of the promoter region can inhibit the binding of TFs, thereby leading to lighter colored fruits in some varieties [11]. Conversely, inhibitors of DNA methylation, such as 5-azacytidine (5-aza), can block the action of DNA methyltransferase, thus promoting the regulatory effects of TFs [12].

Among plants in the genus*Citrus*, carotenoids and flavonoids tend to be the predominant pigmented substances [13], and the blood orange (*C. sinensis*L. Osbeck) is the only anthocyanin-rich*Citrus*fruits grown on a large scale [14,15], of which ‘Tarocco’ is the most widely planted blood orange variety in China. Tarocco has been reported to have originated from a spontaneous bud mutation in a Sanguinello orchard during the early 1900s in Siracusa Province, Sicily [15], and it has been established that the molecular basis of the blood orange pigmentation involves the retrotransposon-mediated transcriptional activation of *CsRuby*(a MYB-like TF) [14]. In the ‘Moro’, another blood orange variety, the sequence upstream of this TF has been found to contains the 3ʹ-LTR portion of the TCS1 retrotransposon [14]. Despite no differences in the coding sequences of*Ruby*among multiple Tarocco and Sanguinello clones, considerable variations in the degrees of anthocyanin pigmentation were found in the pulp and peel [15], ranging from a few red vesicles to a deep reddish-purple coloration [16].

As experimental material in this study, we focused on a bud variety discovered in a Tarocco orchard in Wenzhou city of China, which was characterized by a lack of accumulated anthocyanin in the peel and pulp (Fig. S1A). Unlike Tarocco fruits, which typically display a distinct purplish-red color at maturity and after 30 days of postharvest storage at low temperature, the fruits of this variant remained orange-yellow color (Fig. S1B, C). The two varieties have similar flowering periods. In addition, compared with Tarocco plants, the margins of the bud variant leaves were crinkled (Fig. S1D). Notably, these variant traits were established to be stably inherited in the asexually reproduced plants. The aberrant fruit color trait is an established trait of the original ancestor’s and can thus be cited as an example of revertive inheritance. In contrast, the leaf-crumpling trait is not observed in sweet oranges, suggesting that this trait may have resulted from new genetic variation.

In this study, we collected the fruits of*C. sinensis*cv. Tarocco (WT) and its bud variant*C. sinensis*cv. Ouya (MT) at different developmental stages to examine trait changes including fruit size and the contents of anthocyanins, soluble sugars, and organic acids. Moreover, we screened the core genes based on RNA-seq analysis to validate their functionality. In addition, we treated MT fruits with 5-aza and analyzed the expression and methylation levels of the core TFs and structural genes based on RNA-seq and whole-genome bisulfite sequencing (WGBS) procedures. Our findings provide valuable insights into the molecular mechanisms underlying the low anthocyanin content in MT fruits, and also provide support for the promotion of MT as a new variety.

## Materials and methods

### Plant material

From September 2022 to January 2023, we collected WT and MT fruits, which were cultivated in a Fengwei orchard in Wenzhou city (120.41°E, 28.02°N). Our project team undertook the formal identification of the Ouya, but neither was deposited in a publicly accessible herbarium. Furthermore, plants derived from MT scions high-grafted with WT as the rootstock were found to have a similar fruiting period to the parent variety. The first samples were collected at 160 days after flowering (DAF), when the fruit surfaces of both varieties had begun to fade to green, and thereafter, samples were obtained at 15-day intervals until 280 DAF, during which time, a total of nine samples were collected, each consisting of 10 fruits.

### Appearance traits

#### Measurement of fruit weights and size

Fruit weight was determined using a DLX-A8 digital balance (DELIXI, Wenzhou, China) with an accuracy of 0.01 g. Fruit size (equatorial diameters and length) was measured using a 3.0 V electronic digital slide gauge (DELIXI, Wenzhou, China) with an accuracy of 0.01 mm.

#### Measurement of peel and pulp color parameters

The colors of the peel and pulp of fruits were determined using a C-300 colorimeter (Minolta, Osaka, Japan). For the peel, four points were uniformly selected on the equatorial plane of each fruit for testing. For the pulp, four points were uniformly selected on the longitudinal plane of the fruit, 2 cm from the peel. Thereby, the L*, a*, and b* data were obtained using a colorimeter. These values were then used to calculate the hue angle degree (H) and citrus color index (CCI) [17].

All the above appearance traits assessed 10 WT and MT fruits. After the test, the peel was separated from the pulp. The albedo was removed from the peel, and the segment wall was removed from the pulp. Both the peel and pulp were immediately frozen in liquid nitrogen and stored at −80 °C.

### Physicochemical determinations

#### Determination of total soluble solid, soluble sugar, organic acid, vitamin C, and total carotenoid contents of fruits

The total soluble solids (TSS) content of fruits was measured using a digital total soluble solids refractometer (PAL-1; Atago, Tokyo, Japan). After the pulp was initially ground with liquid nitrogen, the soluble sugar and organic acid contents were determined by ultra-performance liquid chromatography (UPLC; soluble sugars: UPLC ACQUITY H-Class, Waters, Milford, USA; organic acids: UltiMate 3000, Thermo, Waltham, USA). Extraction and assay methods were based on the B Yang, H Yao, J Zhang, Y Li, Y Ju, X Zhao, X Sun and Y Fang [18]. The sugar-acid ratio was calculated as the ratio of total soluble solids to total organic acids. The vitamin C contents in fruit pulp samples were determined by titrating an aqueous extract of the pulp with a solution of 2,6-dichlorophenol-indophenol dye to a faint pink endpoint [19]. The total carotenoid contents were measured using a plant carotenoid content assay kit (AKPL004M, Boxbio, Beijing, China).

#### Analysis of total anthocyanins and their fractions

The pulp and peel samples were ground into a powder, and 100 mg was weighed and extracted with 0.5 mL of a methanol/water/hydrochloric acid (500:500:1, V/V/V). Then the extract was vortex-mixed for 5 min, ultrasonicated for 5 min, and then centrifuged at 12,000 × g at 4 ℃ for 3 min. The residue was re-extracted by repeating the above steps under the same conditions. The supernatants were collected and filtered through a membrane filter (0.22 μm). The total anthocyanin content in the samples was measured using the pH differential method as described by J Lee, RW Durst and RE Wrolstad [20]. Theabsorbances of the sample extracts were measured at 520 and 700 nm using a spectrophotometer (UV-2550; Shimadzu, Kyoto, Japan). The contents of anthocyanin fractions ofpulp were determined using MetWare (http://www.metware.cn/) based on an LC-MS/MS platform (QTRAP 6500; AB Sciex, Framingham, USA).

Assessments of each of the physicochemical traits were performed using three biological replicates.

### RNA extraction, library construction, sequencing, and data analysis

Total RNA was extracted from the pulp of WT and MT fruits collected at 235, 265, and 280 DAF using an Omini Plant RNA Kit (DNase I, Cwbio, Nanjing, China) and named WT1/MT1, WT2/MT2, and WT3/MT3, respectively. High-throughput sequencing was performed using the Illumina Hiseq 4000 platform (Shanghai Majorbio Bio-pharm Technology Co., Ltd). To ensure data accuracy, each preparation was analyzed using three biological replicates, resulting in 18 independent RNA samples.

Each sequencing library contained 40.8–85.2 million clean reads, alignment with the pomelo reference genome (http://citrus.hzau.edu.cn/index.php,*Citrus sinensis*v3.0), genes with a false discovery rate below 0.05 and absolute fold change ≥ 2 were considered as differentially expressed genes (DEGs). Kyoto Encyclopedia of Genes (KEGG) and Gene Ontology (GO) enrichment analysis and functional annotation methods are referenced from Y You, C Ju, L Wang, X Wang, F Ma, G Wang and Y Wang [21]. TFs analysis and prediction of the reference PlantTFDB [22].

### Weighted gene co-expressed network analysis (WGCNA)

Co-expression networks were constructed using the weighted gene co-expressed network analysis (WGCNA) package (v1.47) in R. WGCNA was performed using Majorbio Bio-pharm Technology as previously described, with minor modifications [23].

Briefly, WGCNA includes network construction, module and gene selection, and the functional analysis of module genes. After removing genes with extremely low expression levels from the samples, 9571 genes were imported intoWGCNA with the following parameters: soft threshold was 12, network type was signed, minKMEtoStay was 0.3, mergeCutHeight was 0.45, and minModuleSize was 50. Regulatory network diagrams were constructed using Cytoscape 3.7.1 [24]. To identify biologically significant modules, module eigengenes were used to calculate correlation coefficients with the sample traits. We used the contents of four highest content anthocyanins, total anthocyanins, soluble sugars (including glucose, fructose, and sucrose), and organic acids (including quinic, malic, and citric acids) as sample traits.

### cDNA synthesis and RT-qPCR

cDNA was synthesized from extracted RNA using Hifair II First Strand cDNA Synthesis SuperMix (Yeasen, Shanghai, China). RT-qPCR analyses were performed using three independent biological and three technical replicates in a QuantStudio 6 thermocycler (Thermo Fisher Scientific, Waltham, USA), with relative gene expression being calculated using the 2^−△△Ct^method. The primers used for RT-qPCR are listed in Table S1.

### Transient expression of*CsRuby*in fruits

To compare the sequences of*CsRuby*in the two varieties, the respective coding sequences of*CsRuby*were PCR amplified from cDNA libraries prepared from WT and MT newly-grown leaves and cloned using a Hieff Clone^®^Plus One Step Cloning Kit (Yeasen, Shanghai, China). To insert the gene into a pBI101 plasmid for overexpression, we conducted recombinase reactions using a Hieff Clone^®^Universal II One Step Cloning Kit (Yeasen, Shanghai, China) following the manufacturer’s instructions.*Agrobacterium*transformed with the gene constructs was grown overnight in liquid Luria–Bertani medium to an optical density (O.D.) of 0.8–1.0, and diluted to a final O.D. of 0.8 in liquid injection medium containing 0.05 M MES, 2 mM Na_3_PO_4_, 0.5% (m/v) d-glucose, and 0.1 mM acetosyringone. For the assessment of transient expression, we injected both sides of MT fruits’ equatorial plane with either the*CsRuby*overexpression vector or a control empty vector.

### 5-Azacytidine treatment and whole-genome bisulfite sequencing analysis

To assess the effects of DNA methylation in fruits, we used the methylation inhibitor 5-aza (Sigma-Aldrich, St. Louis, USA) to treat MT fruits collected at 235 DAF. The 5-aza was dissolved in sterile water to a final concentration of 20 mM and passed through 0.22-µm polyethersulfone filters. Aliquots of approximately 1.0 mL were carefully injected into the equatorial plane of fruits using an HPLC syringe with a needle inserted into the fruits to a depth of approximately 0.3–0.5 cm. One half of each fruit was injected with 5-aza, while the other half was injected with sterile water as a control. The 5-aza and sterile water injections were subsequently repeated three to five times at 2-day intervals. After 7 days of treatment, the fruits were cut into two halves using a scalpel, frozen in liquid nitrogen, and stored at −80 °C until used for analysis. Samples obtained frompulp on both sides of the fruits were subjected to RNA-seq and whole-genome bisulfite sequencing analysis (WGBS). DNA/RNA extraction, library construction, and sequencing were performed by e-Gene (www.egenetech.com).

### Statistical analysis

All data are presented as means ± standard deviation (SD). For each treatment, the mean and SD values were calculated using Microsoft Excel. Subsequently, statistically significant differences between WT and MT samples were determined by a two-tailed Student’s*t*-test (*P*< 0.05).

## Results

### Physicochemical properties

**Fig. 1:**
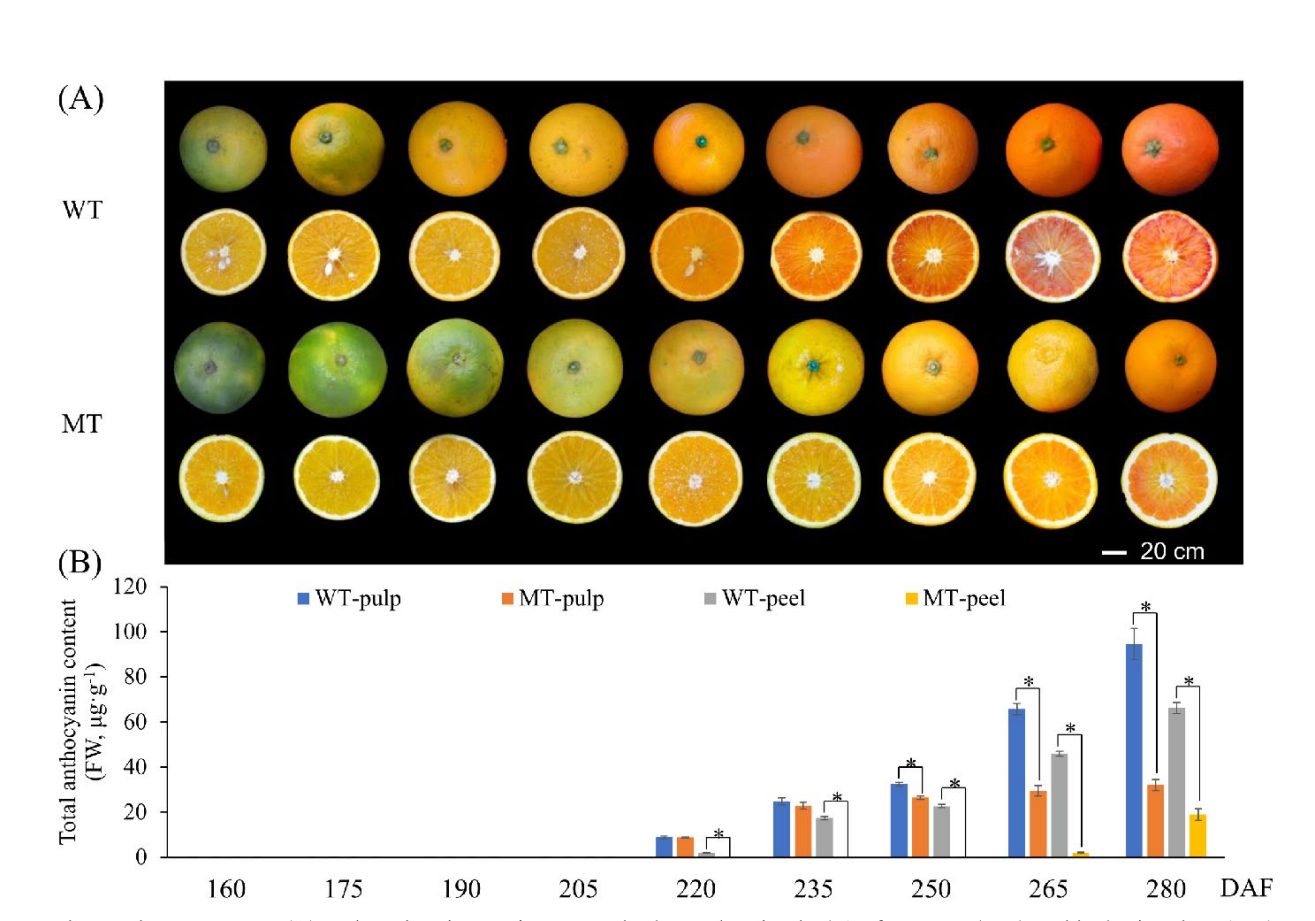
The appearance (**A**) and total anthocyanin content in the peel and pulp (**B**) of Tarocco (WT) and its bud variety (MT) at different development stages. Data are mean ± standard error (*n*= 3 biologically independent replicates). Asterisks indicate significant differences: ∗*P*< 0.05

Between 160 and 235 DAF, the peel of WT fruits was found to be consistently more orange-red in color than that of the MT fruits. In contrast, between 160 and 205 DAF, the pulps of the two varieties were similar in color, and no significant anthocyanin accumulation was observed. It was not until 220 DAF that the color of the MT pulp was significantly paler than that of the WT. At this time,the edges of the WT peel were red. Thereafter, the red coloration in the WT gradually spread to the central axis, and by 280 DAF, it had covered the entire pulp in filaments pattern. In contrast, in the MT, only the edge of the peel remained red (Fig.1A).

The color characteristics of the peeland pulp of the two varieties are shown in Tables S2 and S3, respectively. Specifically, between 160 and 235 DAF, the L* values of MT peel were significantly lower than those of the WT peel. However, no significant differences were detected between the MT and WT peels from 250 to 280 DAF, nor were there significant differences in the pulp throughout all the assessed stages. As the fruits of both varieties continued to ripen, the a* values of the peel and pulp gradually increased. Nevertheless, the a* values in the MT pulp were consistently and significantly lower than those in the WT pulp, and significant differences in the a* values of the peels were observed after 205 DAF. At all stages, the CCI values of the MT peel were significantly lower than those of the WT peel. Specifically, at 280 DAF, the CCI values were 3.69 for the WT peel (characterized by an orange - red appearance) and 2.46 for the MT peel (with an orange appearance). At this time, the CCIs of MT and WT were −1.68 and 1.12, respectively. In contrast to the trends of a*, b*, and CCI, notable differences were observed in the trends of H values. Throughout all sampling stages, the H values recorded for the MT pulp and peel were significantly higher than those for the WT.

Comparisons of the changes in fruit weight, size, TSS, soluble sugar, and organic acid contents in the pulp of WT and MT fruits at different stages revealed that the equatorial diameter of MT fruits was significantly greater than that of WT fruits, resulting in higher fruit weights of MT (254.08 g) compared with those of WT (242.36 g) at 280 DAF (Table S4). The TSS contents of MT were also significantly higher than those of WT. The TSS of MT exceeded 15 °Brix at 190 DAF and then remained between 14.91 and 15.80 °Brix. In contrast, the TSS of WT was only 12.91 °Brix at 190 DAF and gradually increased to 14.81 °Brix at 280 DAF (Table S4). Similarly, the total soluble sugar content of MT between 220 and 265 DAF was significantly higher than those recorded for WT (Fig. S2A). Citric acid was the most abundant among the three assessed organic acids (citric, quinic, and malic acid). In both varieties, the citric acid content gradually increased from 160 to 190 DAF and then declined, and the content in MT was significantly lower than that in WT. Comparatively, there was less variation in the contents of the other two organic acids (Fig. S2B). These differences in sugar and acid contents between MT and WT were reflected in the respective sugar-acid ratios. In both varieties, the sugar-acid ratios gradually increased with fruit maturation, and the ratios in MT were consistently significantly higher than those in WT after 190 DAF (Table S4).

In addition, we examined the contents of vitamin C and total carotenoids in the pulp at 280 DAF. The vitamin C contents were 53.70 and 51.01 mg·100 g^−1^in WT and MT, respectively, with no significant difference (Fig. S2C). Compared with MT, the carotenoid levels in WT were significantly higher, with values of 48.28 and 59.62 µg·g^−1^, respectively (Fig. S2D).

### Changes in the contents of total anthocyanins and their fractions

The changes in the contents of total anthocyanins in the peel and pulp of the two varieties were observed to follow trends similar to those of the change in fruit appearance, with no anthocyanins being detected until 220 DAF. As the fruits gradually matured, the contents of total anthocyanins in the pulp tended to remain relatively low, and no significant differences were detected at 220 and 235 DAF. However, compared with MT fruits, significantly higher levels of total anthocyanins were detected in the peel of WT fruits. From 250 to 280 DAF, the total anthocyanins in both the pulp and peel were significantly higher in WT than in MT (Fig.1B).

**Fig. 2:**
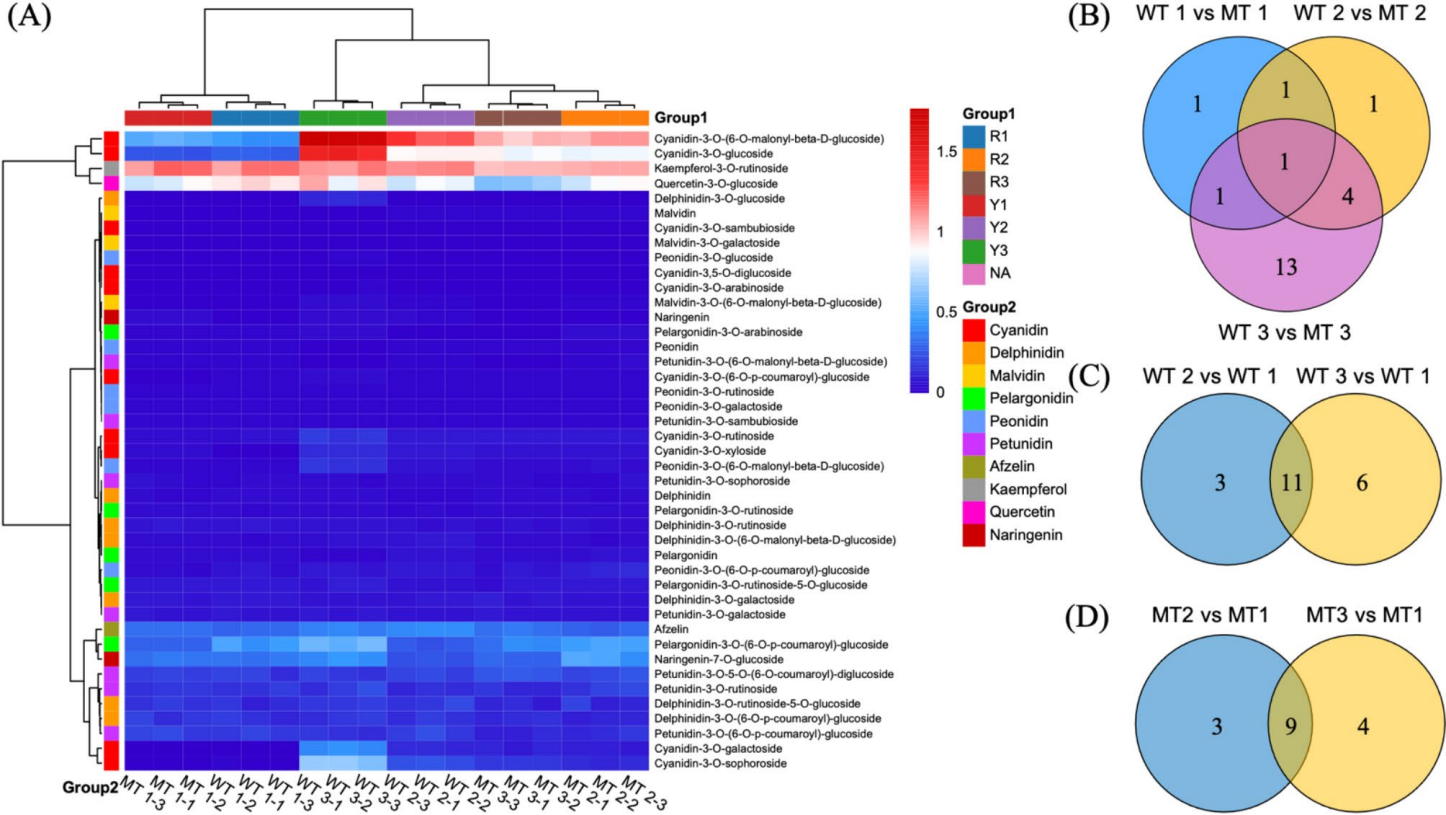
Heatmap (**A**) and venn diagram (**B-D**) of different anthocyanin fractions in the pulp of Tarocco (WT) and its bud variety (MT) at different developmental stages (detailed grouping information in Table S2)

Furthermore, we also analyzed the flavonoid fractions in pulp samples collected at 235, 265, and 280 DAF (Fig.2A), and accordingly detected a total of 38 anthocyanins and 6 flavanones, with the contents of cyanidin-like anthocyanins being relatively high, among which cyanidin-3-O-(6-O-malonyl-beta-d-glucoside) and cyanidin-3-O-glucoside had the highest relative contents. The levels of both these anthocyanins gradually increased with fruit maturation, with significantly higher levels being detected in WT (53.68 and 9.96 µg·g^−1^, respectively) than in MT (27.14 and 6.61 µg·g^−1^, respectively) when measured at 289 DAF. Rutin, kaempferol-3-O-rutinoside, and quercetin-3-O-glucoside were identified as the flavonoids with higher relative contents. At 280 DAF, their levels were again found to be significantly higher in WT (22.2, 12.64, and 8.26 µg·g^−1^, respectively) than in MT (15.04, 9.76, and 3.42 µg·g^−1^, respectively) (Table1, Table S5).

**Table 1:** The major anthocyanin and flavonoid contents of Tarocco (WT) and bud variety (MT) pulp at different sampling periods (µg·g^− 1^)

Compounds	Stage1 (235 DAF)			Stage2 (265 DAF)			Stage3 (280 DAF)	
	MT	WT	MT		WT	MT		WT
Cyanidin-3-O-(6-O-malonyl-beta-D-glucoside)	2.42 ± 0.14*	1.72 ± 0.18	11.19 ± 1.11*		18.18 ± 2.95	9.96 ± 1.12*		53.68 ± 4.43
Cyanidin-3-O-glucoside	0.79 ± 0.06*	0.92 ± 0.06	6.13 ± 0.18*		7.04 ± 0.27	6.61 ± 0.68*		27.14 ± 2.98
Cyanidin-3-O-rutinoside	0.12 ± 0.01	0.13 ± 0.01	0.21 ± 0.00		0.20 ± 0.01	0.20 ± 0.01*		0.48 ± 0.05
Cyanidin-3-O-sophoroside	0.03 ± 0.00*	0.04 ± 0.01	0.34 ± 0.04*		0.72 ± 0.06	0.48 ± 0.01*		3.46 ± 0.33
Cyanidin-3-O-galactoside	0.01 ± 0.00*	0.01 ± 0.01	0.24 ± 0.03		0.32 ± 0.04	0.26 ± 0.05*		1.60 ± 0.10
Delphinidin-3-O-rutinoside-5-O-glucoside	0.47 ± 0.07*	0.36 ± 0.11	0.38 ± 0.19*		0.54 ± 0.14	0.28 ± 0.06*		0.49 ± 0.05
Delphinidin-3-O-(6-O-p-coumaroyl)-glucoside	0.48 ± 0.13	0.43 ± 0.14	0.27 ± 0.03*		0.42 ± 0.10	0.34 ± 0.06*		0.46 ± 0.09
Pelargonidin-3-O-(6-O-p-coumaroyl)-glucoside	0.95 ± 0.03*	1.93 ± 0.32	2.08 ± 0.13*		0.82 ± 0.09	1.39 ± 0.23*		2.71 ± 0.17
Petunidin-3-O-rutinoside	0.46 ± 0.06	0.46 ± 0.06	0.56 ± 0.13*		0.36 ± 0.05	0.37 ± 0.08*		0.56 ± 0.19
Petunidin-3-O-5-O-(6-O-coumaroyl)-diglucoside	0.55 ± 0.03	0.52 ± 0.12	0.70 ± 0.08*		0.61 ± 0.04	0.82 ± 0.05*		0.50 ± 0.10
Afzelin	1.12 ± 0.04*	1.02 ± 0.04	1.59 ± 0.07*		0.97 ± 0.06	1.19 ± 0.10*		1.42 ± 0.09
Kaempferol-3-O-rutinoside	14.31 ± 2.57	13.44 ± 2.39	12.96 ± 0.74*		10.66 ± 0.16	9.76 ± 0.23*		12.64 ± 1.48
Quercetin-3-O-glucoside	5.71 ± 0.96*	7.83 ± 0.58	5.75 ± 0.76		6.19 ± 1.08	3.42 ± 0.34*		8.26 ± 2.38
Naringenin-7-O-glucoside	1.23 ± 0.05	1.21 ± 0.12	0.76 ± 0.03*		2.03 ± 0.36	0.97 ± 0.04*		1.66 ± 0.20
Rutin	42.32 ± 8.29	40.51 ± 10.06	31.19 ± 6.11*		18.64 ± 3.57	15.04 ± 2.99*		22.20 ± 1.75

Data are mean ± standard error (*n* = 3 biologically independent replicates). The asterisks indicate statistically significant differences determined by the Student’s *t*-test, *P* < 0.05

A comparison of the number of anthocyanins and flavonoids among the different groups revealed four differential metabolites between the WT and MT fruits at 235 DAF, with the number reaching 7 and 19 at 265 and 280 DAF, respectively, of which only the difference in cyanidin-3-O-(6-O-malonyl-beta-d-glucoside) was significant at all assessed time points (Fig.2B). Among the differential metabolites detected in the same variety at 265 and 280 DAF compared with 235 DAF, a majority (11 and 9 metabolites) were commonly differentially synthesized in WT and MT, respectively (Fig.2C, D).

### RNA-seq of WT and MT fruits during different developmental stages

To investigate the mechanisms associated with the differential synthesis of anthocyanins in WT and MT at the gene expression level, we also conducted RNA-seq analysis of samples in which anthocyanin fractions were detected. An overview of the transcriptome data (including quality check information) is shown in Table S6.

Correlations analysis between samples demonstrated good reproducibility among the three biological replicates, with the least variability between WT1 and WT2, while relatively large differences among WT1, MT3, MT1, and WT3 (Fig. S3A). Principal component analysis (PCA) of the WT and MT groups at the three time points was performed to examine the variation in data across different groups. The first principal component (PC1) accounted for 27.80% of the observed variance, suggesting that gene expression profiles were influenced by the degree of fruit development. In contrast, the second principal component (PC2) accounted for only 15.37% of the variance (Fig. S3B). Interestingly, there was less variability between WT and MT in the subsequent stage (i.e., WT1 vs. MT2 and WT2 vs. MT3).

In addition to the 49,567 annotated genes in the reference genome, we discovered 1,695 previously unidentified genes (see Table S7 for detailed information). Three comparison groups (WT1 vs. MT1, WT2 vs. MT2, and WT3 vs. MT3) were established for the two varieties at different time points (Fig. S3C). A total of 321 differentially expressed genes (DEGs) (207 up-regulated and 114 down-regulated) were identified at 235 DAF, whereas 734 (617 up-regulated and 117 down-regulated) and 816 (507 up-regulated and 309 down-regulated) DEGs were identified at 265 and 280 DAF, respectively. This indicates that the number of DEGs between the two varieties gradually increased with fruit maturation.

Details of the expression levels and statistical data for TFs in the DEGs are presented in Table S8. Notably, five common TFs detected in WT and MT, namely Cs_ont_1g026270 (*CsATHB40*), Cs_ont_1g027600 (*CsBBX18*), Cs_ont_3g011170 (*CsLBD1*), Cs_ont_5g001230 (*CsUNE10*), and Cs_ont_6g005110 (*CsRuby*), showed significant differences in expression throughout all the assessed stages. This suggests that these TFs may be involved in regulating the differential accumulation of flavonoids in the fruits of the two varieties. Among these,*CsBBX18*,*CsUNE10*, and*CsRuby*are B-box, bHLH, and MYB class TFs, respectively, which may be associated with flavonoid biosynthesis.

### KEGG and GO enrichment analysis of DEGs

**Fig. 3:**
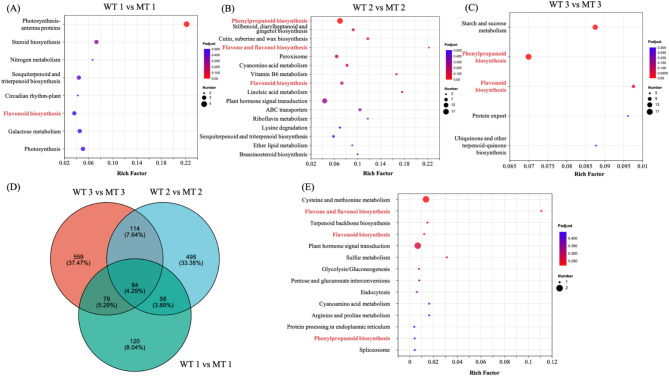
Identification and functional characterization of the differentially expressed genes (DEGs) between Tarocco (WT) and its bud variety (MT) pulp samples. KEGG enrichment analysis of the DEGs between (**A**) WT 1 vs. MT 1, (**B**) WT 2 vs. MT 2, and (**C**) WT 3 vs. MT 3, (**D**) Venn diagram depicting the shared and specific genes between the three compared groups of pulp samples, (**E**) KEGG analysis of 64 DEGs common to the three compared groups

Enrichment analysis of the DEGs obtained from the three comparison groups was conducted with reference to the KEGG and GO databases. For all three comparison groups, KEGG analysis revealed an enrichment of DEGs in the flavonoid biosynthesis pathway. As the fruit matured, both the upstream and downstream pathways of flavonoid biosynthesis were concurrently enriched in phenylpropanoid biosynthesis, as well as the biosynthesis of flavonoids and flavonols (Fig.3A-C).

In addition, both KEGG and GO analyses showed an enrichment of photosynthesis-related categories and pathways, including photosynthesis-antenna proteins, photosystem I, photosynthesis, and light harvesting, in WT1 vs. MT1. For the WT2 vs. MT2 comparison, DEGs were found to be enriched in pathways associated with cellular transporters, such as ABC transporters and secondary active transmembrane transporter activity. In the case of the WT3 vs. MT3 comparison, GO analysis revealed enrichment in the categories associated with anthocyanin synthesis, including phenylalanine ammonia-lyase activity and flavonoid biosynthesis (Fig. S4A-C).

To identify specific genes associated with WT and MT at different time points, we constructed Venn diagrams for the DEGs. As shown in Fig.3D, we identified 64 DEGs common to the three groups, suggesting that these core conserved genes may be associated with the developmental traits of both varieties. Subsequently, we carried out KEGG and GO enrichment analysis on these 64 DEGs, with the former revealing an enrichment of flavonoid biosynthesis, phenylpropanoid biosynthesis, and flavone and flavonol biosynthesis pathways (Fig.3E), and the latter indicating enrichment of five methylation-related DEGs. All of these DEGs were found to be more highly expressed in WT than in MT (Fig. S4D).

### Identification of WGCNA modules associated with anthocyanin and fruit quality traits

To ensure the accuracy of network construction, we initially removed genes with extremely low expression levels in all samples (for details, see the Materials and Methods). A gene clustering tree was constructed by consideration the correlations between gene expression levels. Gene modules were established based on clustering relationships; that is, genes with similar expression patterns were grouped into the same module. The modules were generated by cutting off branches from the dendrogram. Finally, similar modules were merged to obtain seven modules, as shown in Fig. S5A-C, which were clustered into three main branches (three, two, and two modules, respectively; Fig. S5D).

**Fig. 4:**
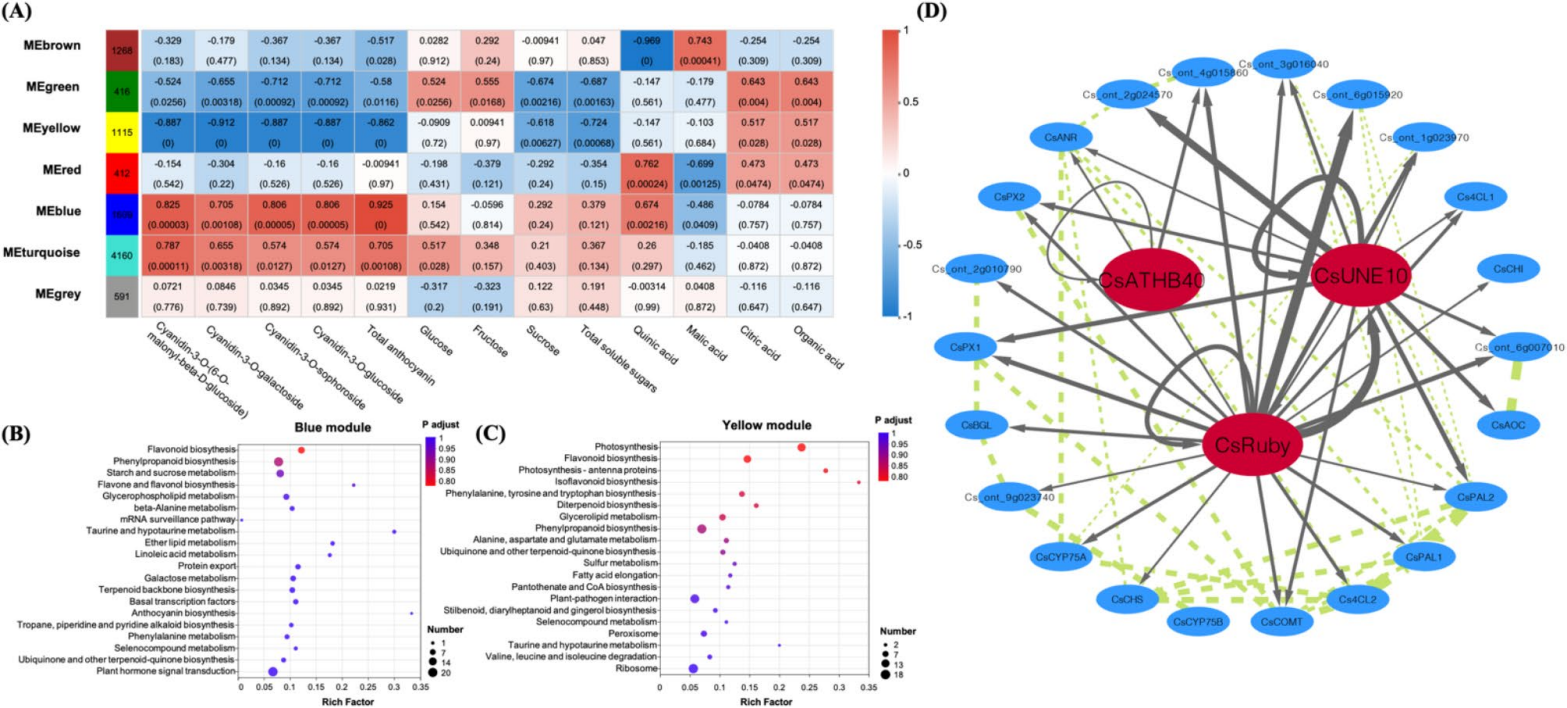
Weighted gene co-expressed network analysis (WGCNA)-based grouping of 9571 genes based on the performance of 13 traits (including different fractions of anthocyanins, soluble sugars, and organic acids). (**A**) The left panel shows the 7 modules and the number of genes in each module. Color indicates the correlation coefficient of a specific module and the treatment, as shown in the right. KEGG of genes in the positive and negative modules blue (**B**) and yellow (**C**) with the highest correlation with total anthocyanin traits. (**D**) Transcriptome co-expression network analysis, transcription factors and structural genes involved in the modules screened by WGCNA, gray arrows and green dotted lines indicate the regulation between the transcription factors and target genes and the interactions between proteins, respectively, and the thickness of the line segments indicates the high relevance of the interactions

Genes associated with anthocyanin synthesis can be better categorized based on WGCNA. We found that both sucrose and total soluble sugars were significantly negatively correlated with the yellow and green modules, whereas citric acid and organic acids were positively correlated with these two modules. In addition, the brown module showed significant positive and negative correlations with quinic and malic acids, respectively, while the red module exhibited different correlation patterns. The turquoise module showed significant positive correlations only with cyanidin-3-O-(6-O-malonyl-beta-d-glucoside) and total anthocyanins (Fig.4A). Both total anthocyanins and four specific anthocyanin components were highly correlated with the blue and yellow modules. The blue module was positively correlated and enriched for several anthocyanin synthesis-related metabolic pathways, including flavonoid biosynthesis, phenylpropanoid biosynthesis, flavone and flavonol biosynthesis, anthocyanin biosynthesis, and phenylalanine metabolism (Fig.4B), whereas the latter was negatively correlated, it was also enriched for several anthocyanin synthesis-related pathways, as well as photosynthesis-related pathways such as photosynthesis and photosynthesis-antenna proteins (Fig.4C).

Genes associated with anthocyanin synthesis can be better categorized based on WGCNA. We found that both sucrose and total soluble sugars were significantly negatively correlated with the yellow and green modules, whereas citric acid and organic acids were positively correlated with these two modules. In addition, the brown module showed significant positive and negative correlations with quinic and malic acids, respectively, whereas the red module showed different correlations. The turquoise module showed significant positive correlations only with cyanidin-3-O-(6-O-malonyl-beta-d-glucoside) and total anthocyanins (Fig.4A). Both total anthocyanins and four specific anthocyanin components were highly correlated with the blue and yellow modules. The blue module was positively correlated and enriched for several anthocyanin synthesis-related metabolic pathways, including flavonoid biosynthesis, phenylpropanoid biosynthesis, flavone and flavonol biosynthesis, anthocyanin biosynthesis, and phenylalanine metabolism (Fig.4B). In contrast, the yellow module was negatively correlated but also enriched for several anthocyanin synthesis-related pathways, as well as photosynthesis-related pathways such as photosynthesis and photosynthesis-antenna proteins (Fig.4C).

All TFs found to show a differential expression between the WT and MT were categorized into different modules, with blue having a progressively higher number of differentially expressed TFs in the three stages (12, 15, and 33, respectively), while the total numbers and differences in the yellow module were smaller (2, 0, and 2, respectively). Interestingly, all five TFs identified through enrichment analyses were found in the blue module (Table S8), thereby tending to indicate that certain DEGs in the blue module may be key factors contributing to the differential accumulation of anthocyanins in WT and MT. Accordingly, we selected these five TFs as hub genes and aimed to determine their regulatory associations with flavonoid synthesis-related genes in the blue module, among which only*CsATHB40*,*CsUNE10*, and*CsRuby*can act as regulatory proteins (Table S9). All of these can mediate self-transcriptional regulation, and*CsUNE10*and*CsRuby*can regulate each other. Moreover, we found that they can also participate in the regulation of the flavonoid synthesis-related genes in the blue module, including*CsPAL1*(Cs_ont_6g020620),*CsPAL2*(Cs_ont_8g005310),*CsAOC*(Cs_ont_5g045250),*Cs4CL1*(Cs_ont_6g025120),*Cs4CL2*(Cs_ont_5g016610),*CsCHS*(Cs_ont_9g012610),*CsCHI*(Cs_ont_7g004690), and*CsCOMT*(Cs_ont_3g008120) (Fig.4D).

### Combined transcriptomic and flavonoid analysis

Among the MBW complexes comprising important TFs that regulate anthocyanin synthesis, we found that only the expression levels of*CsRuby*showed a trend similar to that of total anthocyanin content. In all three stages, the expression of*CsRuby*in WT fruits was significantly higher than that in MT fruits, and its expression gradually increased with fruit maturation. In contrast, the expression pattern of*CsTTG1*(a WD40 TF) was opposed to that of*CsRuby*. The expression of*CsAN1*(a bHLH TF) and another*CsWD40*was significantly higher in MT than in WT at 265 DAF, but diametrically opposite at 280 DAF (Fig. S6).

Among the structural genes, with the exception of*CsPAL*(Cs_ont_7g006400, Cs_ont_8g005310, and Cs_ont_6g020620) and*Cs4CL*(Cs_ont_5g016610 and Cs_ont_6g025120), none of those in the phenylpropanoid pathway were significantly up-regulated in MT compared with WT. Comparatively, all genes of the flavonoid biosynthesis pathway (including*CsCHS*,* CsCHI*,* CsF3H*,* CsF3’H*,* CsF3’5’H*,* CsDFR*,* CsANS*, and*CsBZ1*) showed significantly lower expression in MT than in WT. Furthermore, among the genes in the anthocyanin biosynthesis pathway (including*Cs3MaT1*,*Cs3GGT*, and*CsUDPG*), only*Cs3MaT1*(Cs_ont_2g020750) and*CsUDPG*(Cs_ont_2g015530 and Cs_ont_2g022650) exhibited a trend similar to that of genes associated with the flavonoid biosynthesis pathway, whereas other genes showed no significant differences between the two varieties (Fig. S6).

Collectively, these findings provide evidence to indicate that in WT,*CsRuby*enhances the anthocyanin content in fruits by regulating the expression of gene associated with the flavonoid biosynthesis pathway, whereas this process appears to be suppressed in MT.

To assess whether the expressed transcripts were correctly quantified, we analyzed 11 transcripts (three TFs and eight structural genes associated with flavonoid synthesis), and accordingly found that the relative expression and TPM of each gene was significantly correlated (Fig. S7), thereby confirming the results of RNA-seq analysis.

### Functional validation of*CsRuby*

The coding sequences of*CsRuby*from MT and WT were cloned and compared with those of the Navel (JN402329), Moro (JN402330.1), and Jingxian (JN402333) varieties, all of which are registered in the NCBI database and are identical (Fig. S8A). In addition, the coding sequence of*CsRuby*cloned from MT and overexpressed in MT pulp, it significantly promoted the accumulation of anthocyanins and the expression of related synthetic genes in the pulp (Fig. S8B-E).

### Changes in gene expression and DNA methylation in response to 5-aza treatment

**Fig. 5:**
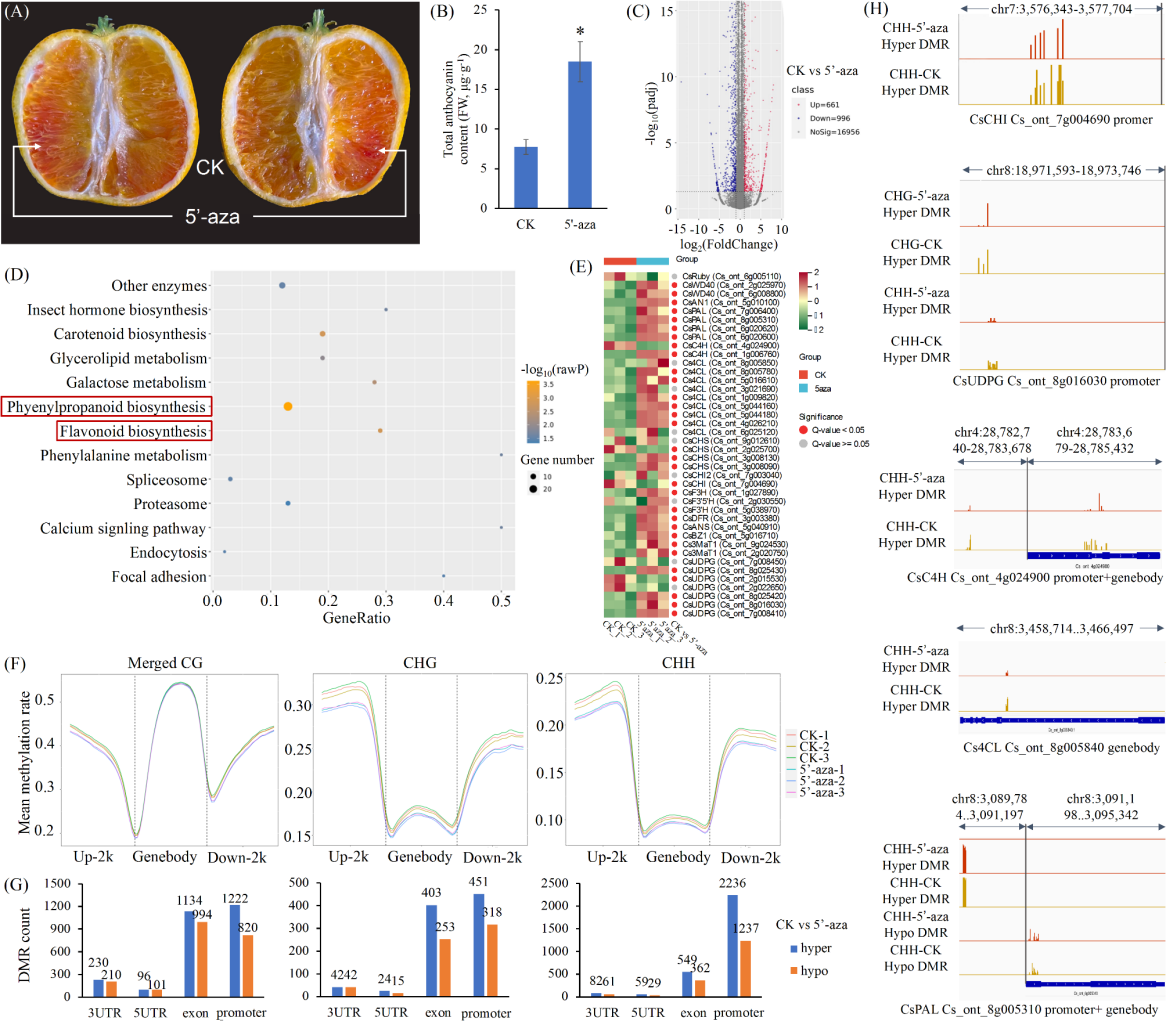
Changes in the anthocyanin content, gene expression, and methylation levels based on whole-genome bisulfite sequencing (WGBS) analysis in bud variety (MT) after 5-aza treatment.**A**and**B**, changes in pulp phenotype and anthocyanin content after 5-aza treatment.**C**, differentially expressed genes (DEGs) in 5-aza and CK pulp were analyzed using RNA-seq.**D**, KEGG enrichment analysis of these DEGs.**E**, the expression levels of anthocyanin-related genes in pulp after 5-aza and CK treatments.**F**, DNA methylation landscape of merged CG, CHG, and CHH (including gene bodies and ~ 2-kb upstream and downstream regions).**G**, differentially methylated region (DMR) count of merged CG, CHG, and CHH (3UTR, 5UTR, exon, and promoter).**H**, visualization of the methylation levels in the anthocyanin-related genes (including *CsCHI*, *CsUDPG*, *CsC4H*, *Cs4CL*, and *CsPAL*) promoter and gene body region. Hyper, hypermethylated; Hypo, hypomethylated

After treatment with 5-aza, a significant accumulation of anthocyanin was detected in the pulp of MT fruits (Fig.5A, B). By comparing the RNA-seq of the CK and 5-aza treated samples, a total of 1657 DEGs were identified between the two treatments. Among them, 661 and 996 were up- and down-regulated, respectively (Fig.5C). KEGG enrichment analysis of these DEGs revealed an enrichment of phenylpropanoid biosynthesis and flavonoid biosynthesis, two pathways associated with anthocyanin synthesis (Fig.5D). However, the flavonoid biosynthesis pathway was enriched with both up- and down-regulated genes, the phenylpropanoid biosynthesis pathway was enriched only with down-regulated genes, as was the carotenoid biosynthesis pathway (Fig. S9A, B). In addition, GO enrichment analysis revealed that the categories of categories chitin and external encapsulating structure synthesis were enriched with up-and down-regulated genes, respectively (Fig. S9C, D). Furthermore, the levels of*CsPAL*,*CsC4H*,*Cs4CL*,*CsCHS*,*CsF3H*,*CsF3’5’H*,*CsF3’H*,*CsDFR*, and*CsANS*, along with numerous other structural genes associated with anthocyanin synthesis, as well as TFs such as*CsWD40*and*CsAN1*, were significantly higher after 5-aza treatment. Nevertheless, no significant changes in the expression of*CsRuby*were detected in response to this treatment (Fig.5E).

We performed WGBS for the 5-aza treatment and CK groups and obtained > 50.93 million clean reads (6.63 Gb) from each sample, among which at least 77.00% mapped to the reference genome (Table S10). A comparison of the DNA methylation dynamics patterns between the CK and 5-aza treatment samples showed that the proportions of CG and CHG methylation were higher in the CK group, at 12.94% and 15.52%, respectively, whereas that the proportions of CHH methylation were relatively higher in the 5-aza treated group, at 73.15% (Fig. S10A).

Furthermore, in all CG, CHG, and CHH methylation contexts, reductions in DNA methylation were detected within the 5ʹ and 3ʹ regions (in the upstream or downstream 2 K sequences) of genes in the 5-aza treated samples compared with the CK samples, especially in the case of CHG and CHH methylation (Fig.5F, S10B-D).

Analysis of the differentially methylated regions (DMR) between the CK and 5-aza groups revealed a higher number of hyper-DMRs compared with hypo-DMRs in all contexts of CG, CHG, and CHH, with the most pronounced effects detected in promoters and exons (Fig.5G). KEGG analysis of DMRs indicated an enrichment of the flavonoid biosynthesis pathway in the CG, CHG, and CHH contexts (Fig. S11).

In this case, the promoters of CsCHI, CsUDPG, CsC4H, and CsPAL were characterized by a reduction in CHH methylation levels following 5-aza treatment, and similar reductions were detected in the gene body regions of CsC4H, Cs4CL, and CsPAL (Fig.5H). In addition, three and one differentially methylated cytosines were identified in the introns and exons of CsRuby, respectively (Fig. S12).

## Discussion

### Differences in the traits of MT and WT fruits

Currently, numerous new*citrus*varietiesare derived from natural mutation [25], and a majority of the*citrus*varieties validated, registered, and granted plant variety rights in China are selected by bud mutation (i.e., somatic variation) [26]. However, in most cases, somatic variations result in new traits that may not be superior to those of the original varieties; moreover, some of these traits may not be apparent after subsequent grafting [27,28].

Consequently, in this study, we performed high grafting of the MT and WT varieties, and accordingly found that the resulting plants of two varieties still showed significant differences in appearance and fruit quality. In terms of fruit quality traits, MT was superior to WT in terms of fruit weight, size, and the contents of soluble sugars and organic acids. MT fruits had high sugar and low acid content, which is more in line with the preferences of East Asian consumers [29]. In terms of flavonoids, the contents of different types of anthocyanins in MT fruits were significantly lower than those in WT fruits. However, there were no significant differences between the two varieties in other flavonoids (e.g., rutin and kaempferol-3-O-rutinoside). Based on the analysis of samples collected at different time points, we established thatMT fruits accumulated soluble sugars more rapidly and reduced organic acids faster, reaching the ripening and harvestable standard between 190 and 205 DAF, whereas WT fruits could not be harvested until between 250 and 280 DAF [30].

In this regard, whereas numerous studies have demonstrated that high-sugar and high-acid fruit environments are more conducive to anthocyanin accumulation [31–33], we found that in MT fruits, which have relatively high sugar and low acid contents, anthocyanins accumulate at a slower rates in both the peel and pulp compared with WT fruits. Accordingly, we postulate that the suppression of anthocyanin accumulation in MT is due to the inhibition of a “switch” that regulates anthocyanins. Furthermore, the lower pulp pH may also affect anthocyanin synthesis. However, the effects of these factors need further investigation.

Compared with the WT fruits, MT also had significantly lower levels of total carotenoids, whereas we observed no significant differences between the two varieties in the content of vitamin C, the most representative nutrient in blood oranges [16]. Based on these findings, we identified MT as a new low-anthocyanin blood orange variety that can ripen up to 1 month earlier than the Tarocco variety traditionally cultivated in Wenzhou City. Moreover, the MT variety produces larger fruits with a more favorable sugar–acid ratio, and thus could contribute to the promotion of blood orange production in China.

### Effects of the hypothetical “switch” on the synthesis of anthocyanins

Among*Citrus*fruits, blood oranges are among the few varieties of orange that accumulate comparatively large amounts of anthocyanins in ripe fruits [15,34]. Therefore, bud-growth materials with differential anthocyanin accumulation are ideal for investigating the mechanisms of anthocyanin synthesis in*Citrus*[35]. As in other plants, the anthocyanin synthesis pathway consists of three main stages: the phenylpropanoid pathway; the synthesis of flavanones, flavones, and flavonols; and, finally, the formation of different anthocyanidins via glycosylation [36]. In this study, we found that the expression of certain genes in the phenylalanine pathway, all genes in the flavonoid biosynthesis pathway, and a few genes in the anthocyanin synthesis pathway were significantly higher in WT fruits than in MT fruits. Given that we established that the differences in the expression of a large number of genes during anthocyanin synthesis were not related to changes in the structural genes of a particular step, we hypothesize that these changes may be mediated by certain TFs that play key regulatory roles in this process [37,38].

The findings of previous studies have indicated that*MYB*TFs, as well as other TFs such as*BBX*, may play key regulatory roles in anthocyanin synthesis [5,39]. Accordingly, we examined the activity of five selected TFs at all three assessed stages of fruit maturation. Notably, it has been established that the promoter of*CsRuby*contains a copia-like retrotransposon insertion that enables it to regulate blood orange anthocyanin production [34]. We detected no differences in the coding sequences of*CsRuby*in MT and WT, in which it can promote anthocyanin synthesis. However, whether these are differences in the promoter or other regions of this gene between the two varieties needs further investigation.

Among the other TFs assessed, CsTTG1 and CsWD40 (WD40), and CsAN1 (bHLH), which can form MBW complexes with CsRuby, have been shown to be jointly involved in the regulation of anthocyanin synthesis in plants [40,41]. However, in*citrus*, members of this complex also interacts with each other [42], and CgAN1 can synergistically interact with CgRuby to promote anthocyanin accumulation [31], although the functions of the homologous CsTTG1 and CsAN1 proteins in anthocyanin synthesis remain unclear [32,43].

In the present study, we found that neither of these proteins had a positive effect on anthocyanin synthesis. That is, for most of the assessed stage, there were no significant differences in the expression of these TFs between the two varieties.*BBX18*has been established to regulate thermoresponsive growth via the PRR5-PIF4 pathway, in which PIF4 inhibits anthocyanin synthesis [44]. The sequence of UNE10, a member of the PIF family, contains a domain similar to that of PIF4 [45], and whether it is involved in the regulation of BBX18, and thus affects anthocyanin accumulation is an interesting direction for future research. In addition, LBD proteins have been demonstrated to regulate anthocyanin and nitrogen metabolism, and respond to phytohormones and environmental stimuli during organ boundary formation [46], among which ATHB has been established to play pivotal roles in regulating the responses to abiotic stress [47]. Accordingly, it is conceivable that these TFs may also function as key factors that influence anthocyanin synthesis in MT.

### DNA methylation influences the synthesis of anthocyanin

DNA methylation has been shown to play important regulatory roles in fruit development and color formation by modulating the expression of relevant genes [48]. For example, the red and green stripe traits in apples are due to differences in methylation of the promoter regions of MdMYB10 and MdMYB1, TFs that regulate pericarp anthocyanin synthesis, leading to differences in the expression of these genes in different parts of the pericarp [49,50]. In*Citrus*, hypomethylation of the promoter of*AbRuby2*from oyster shell thorns has been found to promote anthocyanin accumulation in pigmented leaves [51]. Similarly, the fungus can induce anthocyanin accumulation, and cause hypomethylation in the promoter region of CsRuby [52]. In the present study, among the 64 genes that were commonly differentially expressed in the three-stage comparison groups, we identified five genes that are associated with methylation i.e., the modification of anthocyanin methylation and the promotion of DNA methylation, thereby providing evidence to indicate that methylation potentially influences anthocyanin accumulation. Furthermore, treatment of MT fruits with 20 mM 5-aza was observed to induce hypomethylation of the promoters and gene bodies of CsC4H and CsPAL, thereby promoting a significant anthocyanin accumulation in the pulp, which is similar to previous finding reported for peach and pear fruits injected with 5-aza at concentrations of 1 and 50 mM, respectively [53,54]. Notably, contrasting findings have been reported for strawberries, where treatment with 1 mM 5-aza inhibited anthocyanin and pigment accumulation in orange callus tissue [55,56]. These findings suggest that as an epigenetic modification, DNA methylation may be subjected to differential regulation, thereby presenting interesting avenues for further research.

**Fig. 6:**
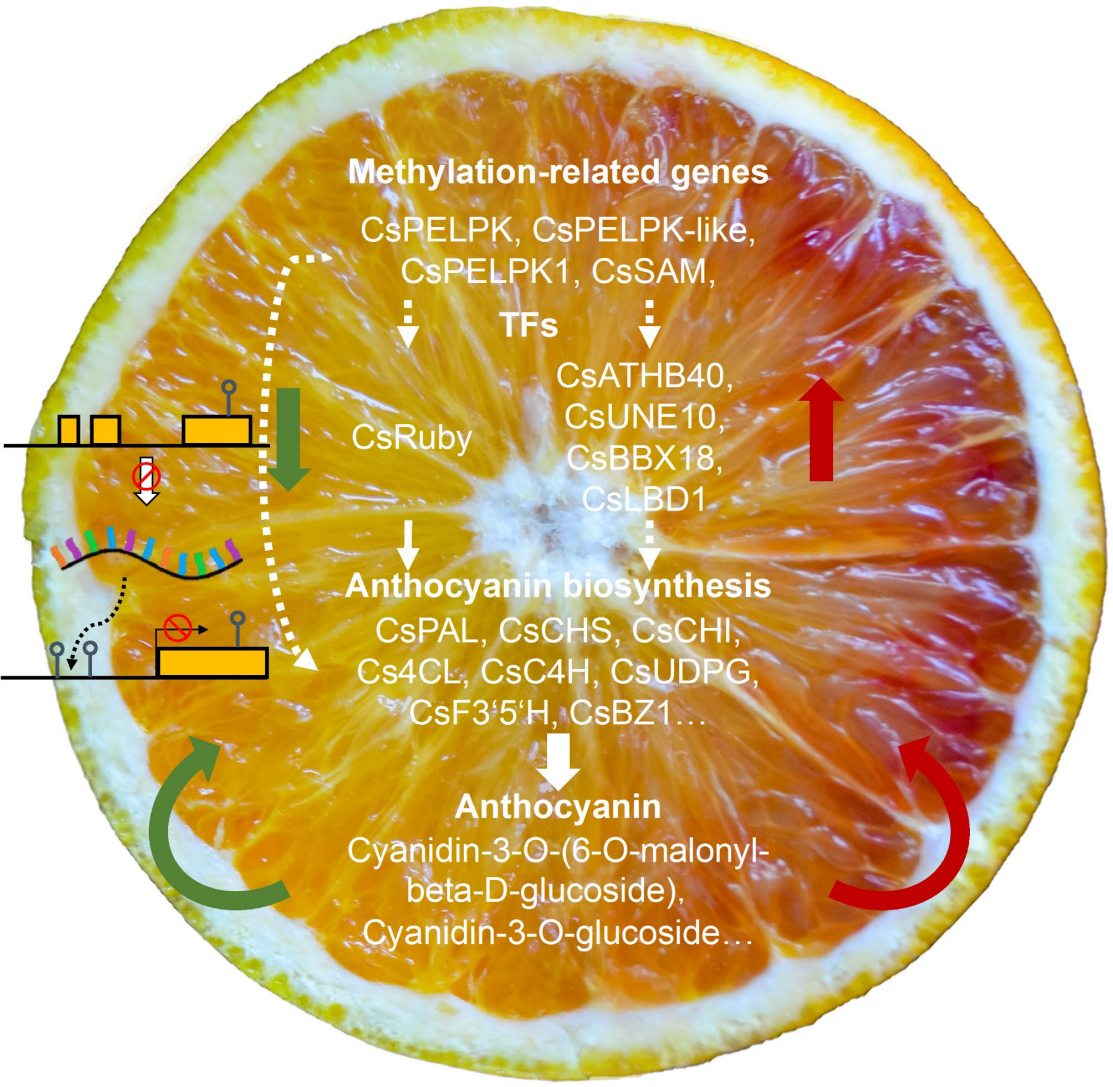
The comparison of blood orange fruit color change mechanisms. Models of possible mechanisms of fruit color change for Tarocco and its bud variety. The inhibition of the expression of CsRuby, a key transcription factor in the anthocyanin synthesis pathway, led to the inhibition of the expression of genes in the anthocyanin biosynthesis pathway. This led to the suppression of the expression of genes in the anthocyanin biosynthesis pathway, which ultimately led to the suppression of the synthesis and accumulation of major anthocyanins in the Tarocco fruit. Red and green arrows indicate increases and reductions in expression or synthesis levels, respectively

In summary, by comparing the fruits of two different blood orange varieties at different development stages, we identified MT as a new variety with less pronounced anthocyanin synthesis, but a more favorable sugar–acid ratio than the WT Tarocco variety. We also identified structural genes associated with anthocyanin synthesis, TFs, and methylation-related genes that we assume are involved in the regulation of flavonoid biosynthesis. Furthermore, by treating MT with 5-aza, we found that the promoters and gene bodies of certain anthocyanin synthesis-related genes showed hypomethylation, and that treated fruit accumulated anthocyanin in the pulp. We accordingly speculate that DNA methylation may play an important role in anthocyanin accumulation in MT. Our findings in this study will provide a valuable basis for further research on the regulation of anthocyanin biosynthesis in*citrus*fruits (Fig.6).

## Electronic Supplementary Material

Below is the link to the electronic supplementary material.

Supplementary Material 1: Figure S1-S12

Supplementary Material 2: Table S1-S9

## Data Availability

MT and WT RNA-seq sequences, and their genome sequencing data can be accessed in the NCBI database using the accession numbers PRJNA1091828.

## Abbreviations

TFs: Transcription factors
DAF: Days after flower
CCI: Citrus color index
TSS: Total soluble solids
DEGs: Differentially expressed genes
SD: Standard deviation
PCA: Principal component analysis
GO: Gene ontology
5-aza: 5-azacytidine
CDS: Coding sequences
WGBS: Whole genome bisulfite sequencing
H: Hue angle degree
UPLC: Ultra-performance liquid chromatography
WGCNA: Weighted gene co-expressed network analysis
DMR: Differentially methylated regions

## References

[1] Chen J, Liu F, Wu RA, Chen J, Wang W, Ye X, Liu D, Cheng H. An up-to-date review: differential biosynthesis mechanisms and enrichment methods for health-promoting anthocyanins of *citrus* fruits during processing and storage. Crit Rev Food Sci Nutr. 2022;64(12):3989–4015. 36322523 10.1080/10408398.2022.2137778

[2] Zhang P, Li Y, Wang T, Cai Z, Cao H, Zhang H, Cao Y, Chen B, Yang D. Statistics on the bioactive anthocyanin/proanthocyanin products in China online sales. Food Sci Nutr. 2021;9(10):5428–34. 34646513 10.1002/fsn3.2500 PMC8498052

[3] Catalano C, Ciacciulli A, Salonia F, Russo MP, Caruso P, Caruso M, Russo G, Distefano G, Licciardello C. Target-genes reveal species and genotypic specificity of anthocyanin pigmentation in *citrus* and related genera. Genes. 2020;11(7):807. 32708660 10.3390/genes11070807 PMC7397085

[4] Ma G, Zhang L, Yamamoto R, Kojima N, Yahata M, Kato M. Molecular characterization of a flavanone 3-hydroxylase gene from *citrus* fruit reveals its crucial roles in anthocyanin accumulation. BMC Plant Biol. 2023;23(1):233. 37131162 10.1186/s12870-023-04173-3 PMC10155330

[5] Bai S, Tao R, Yin L, Ni J, Yang Q, Yan X, Yang F, Guo X, Li H, Teng Y. Two B-box proteins, PpBBX18 and PpBBX21, antagonistically regulate anthocyanin biosynthesis via competitive association with *Pyrus pyrifolia* ELONGATED HYPOCOTYL 5 in the peel of pear fruit. Plant J. 2019;100(6):1208–23. 31444818 10.1111/tpj.14510

[6] Yang L, Chen Y, Wang M, Hou H, Li S, Guan L, Yang H, Wang W, Hong L. Metabolomic and transcriptomic analyses reveal the effects of grafting on blood orange quality. Front Plant Sci. 2023;14:1169220. 37360739 10.3389/fpls.2023.1169220 PMC10286243

[7] Chen J, Liu F, Ismail BB, Wang W, Xu E, Pan H, Ye X, Liu D, Cheng H. Effects of ethephon and low-temperature treatments on blood oranges (*Citrus sinensis* L. Osbeck): anthocyanin accumulation and volatile profile changes during storage. Food Chem. 2022;393:133381. 35691068 10.1016/j.foodchem.2022.133381

[8] Chen J, Zhang Y, Liu F, Chen J, Wang W, Wu D, Ye X, Liu D, Cheng H. The potential of different ripeness of blood oranges (*Citrus sinensis* L. Osbeck) for sale in advance after low-temperature storage: anthocyanin enhancements, volatile compounds, and taste attributes. Food Chem. 2023;417:135934. 36940512 10.1016/j.foodchem.2023.135934

[9] Sicilia A, Scialo E, Puglisi I, Lo Piero AR. Anthocyanin biosynthesis and DNA methylation dynamics in sweet orange fruit *Citrus sinensis* L. (Osbeck) under cold stress. J Agric Food Chem. 2020;68(26):7024–31. 32520546 10.1021/acs.jafc.0c02360 PMC8008385

[10] Sicilia A, Catara V, Scialo E, Lo Piero AR. Fungal infection induces anthocyanin biosynthesis and changes in DNA methylation configuration of blood orange *Citrus sinensis* L. (Osbeck). Plants-Basel. 2021;10(2):244. 33513740 10.3390/plants10020244 PMC7910907

[11] Sun L, Huo J, Liu J, Yu J, Zhou J, Sun C, Wang Y, Leng F. Anthocyanins distribution, transcriptional regulation, epigenetic and post-translational modification in fruits. Food Chem. 2023;411:35540. 10.1016/j.foodchem.2023.135540 36701918

[12] Xu J, Xiong L, Yao J-L, Zhao P, Jiang S, Sun X, Dong C, Jiang H, Xu X, Zhang Y. Hypermethylation in the promoter regions of flavonoid pathway genes is associated with skin color fading during ‘Daihong’ apple fruit development. Hortic Res. 2024;11(3):31. 10.1093/hr/uhae031 PMC10933707 38481937

[13] Lin M, Xu C, Gao X, Zhang W, Yao Z, Wang T, Feng X, Wang Y. Comparative study on secondary metabolites from different *citrus* varieties in the production area of Zhejiang. Front Nutr. 2023;10:1159676. 37252230 10.3389/fnut.2023.1159676 PMC10211264

[14] Butelli E, Licciardello C, Zhang Y, Liu J, Mackay S, Bailey P, Reforgiato-Recupero G, Martin C. Retrotransposons control fruit-specific, cold-dependent accumulation of anthocyanins in blood oranges. Plant Cell. 2012;24(3):1242–55. 22427337 10.1105/tpc.111.095232 PMC3336134

[15] Caruso M, Ferlito F, Licciardello C, Allegra M, Strano MC, Di Silvestro S, Russo MP, Paolo DP, Caruso P, Casas GL, et al. Pomological diversity of the Italian blood orange germplasm. Sci Hort. 2016;213:331–9.

[16] Cebadera-Miranda L, Domínguez L, Dias MI, Barros L, Ferreira I, Igual M, Martínez-Navarrete N, Fernández-Ruiz V, Morales P, Cámara M. Sanguinello and Tarocco (*Citrus sinensis* L. Osbeck): bioactive compounds and colour appearance of blood oranges. Food Chem. 2019;270:395–402. 30174063 10.1016/j.foodchem.2018.07.094

[17] Habibi F, Guillen F, Serrano M, Valero D. Physicochemical changes, peel colour, and juice attributes of blood orange cultivars stored at different temperatures. Horticulturae. 2021;7(9):320.

[18] Yang B, Yao H, Zhang J, Li Y, Ju Y, Zhao X, Sun X, Fang Y. Effect of regulated deficit irrigation on the content of soluble sugars, organic acids and endogenous hormones in Cabernet Sauvignon in the Ningxia region of China. Food Chem. 2020;312:126020. 31874410 10.1016/j.foodchem.2019.126020

[19] Elkhatim KAS, Elagib RAA, Hassan AB. Content of phenolic compounds and vitamin C and antioxidant activity in wasted parts of Sudanese *citrus* fruits. Food Sci Nutr. 2018;6(5):1214–9. 30065822 10.1002/fsn3.660 PMC6060895

[20] Lee J, Durst RW, Wrolstad RE. Determination of total monomeric anthocyanin pigment content of fruit juices, beverages, natural colorants, and wines by the pH differential method: collaborative study. J AOAC Int. 2005;88(5):1269–78. 16385975

[21] You Y, Ju C, Wang L, Wang X, Ma F, Wang G, Wang Y. The mechanism of arbuscular mycorrhizal enhancing cadmium uptake in *Phragmites australis* depends on the phosphorus concentration. J Hazard Mater. 2022;440:129800. 36027745 10.1016/j.jhazmat.2022.129800

[22] Jin J, Tian F, Yang D, Meng Y, Kong L, Luo J, Gao G. PlantTFDB 4.0: toward a central hub for transcription factors and regulatory interactions in plants. Nucleic Acids Res. 2017;45(1):1040–5. 10.1093/nar/gkw982 PMC5210657 27924042

[23] Niu J, Huang Y, Liu X, Zhang Z, Tang J, Wang B, Lu Y, Cai J, Jian J. Single-cell RNA-seq reveals different subsets of non-specific cytotoxic cells in teleost. Genomics. 2020;112(6):5170–9. 32971213 10.1016/j.ygeno.2020.09.031

[24] Han Z, Ahsan M, Adil MF, Chen X, Nazir MM, Shamsi IH, Zeng F, Zhang G. Identification of the gene network modules highly associated with the synthesis of phenolics compounds in barley by transcriptome and metabolome analysis. Food Chem. 2020;323:126862. 32334297 10.1016/j.foodchem.2020.126862

[25] Shimizu T, Kitajima A, Nonaka K, Yoshioka T, Ohta S, Goto S, Toyoda A, Fujiyama A, Mochizuki T, Nagasaki H, et al. Hybrid origins of *citrus* varieties inferred from dna marker analysis of nuclear and organelle genomes. PLoS ONE. 2016;11(11):e0166969. 27902727 10.1371/journal.pone.0166969 PMC5130255

[26] Deng X. A review and perspective for citrus breeding in China during the last six decades. Acta Horticulturae Sinica. 2022;49(10):2063–74.

[27] Wang T, Xiong B, Zheng Z, Qin Z, Deng L, Zheng W, Zhang M, Sun G, He S, Wang J, et al. Natural variation confers ‘Aiyuan 38’ *citrus* mutant a new color and unique flavor. Int J Mol Sci. 2023;24(10):8816. 37240160 10.3390/ijms24108816 PMC10218505

[28] Zhang H, Chen J, Peng Z, Shi M, Liu X, Wen H, Jiang Y, Cheng Y, Xu J, Zhang H. Integrated transcriptomic and metabolomic analysis reveals a transcriptional regulation network for the biosynthesis of carotenoids and flavonoids in ‘Cara Cara’ navel orange. BMC Plant Biol. 2021;21(1):29. 33413111 10.1186/s12870-020-02808-3 PMC7792078

[29] Khairan P, Sobue T, Eshak ES, Kitamura T, Iwasaki M, Inoue M, Yamaji T, Iso H, Tsugane S, Sawada N. Sugary drink consumption and the subsequent risk of gastric cancer: the Japan public health center-based prospective study. Eur J Clin Nutr. 2023;77(2):218–25. 36167978 10.1038/s41430-022-01216-0

[30] Phuangsombut K, Phuangsombut A, Terdwongworakul A. Combination of visible reflectance and acoustic response to improve non-destructive assessment of maturity and indirect prediction of internal quality of red-fleshed pomelo. Int J Food Sci Technol. 2021;56(2):936–44.

[31] Lu ZH, Huang Y, Mao SY, Wu FF, Liu Y, Mao XQ, Adhikari PB, Xu YT, Wang L, Zuo H, et al. The high-quality genome of pummelo provides insights into the tissue-specific regulation of citric acid and anthocyanin during domestication. Hortic Res. 2022;9:175. 10.1093/hr/uhac175 PMC9552194 36238347

[32] Butelli E, Licciardello C, Ramadugu C, Durand-Hulak M, Celant A, Recupero GR, Froelicher Y, Martin C. Noemi controls production of flavonoid pigments and fruit acidity and illustrates the domestication routes of modern *citrus* varieties. Curr Biol. 2019;29(1):158–64. 30581020 10.1016/j.cub.2018.11.040

[33] Durán-Soria S, Pott DM, Osorio S, Vallarino JG. Sugar signaling during fruit ripening. Front Plant Sci. 2020;11:564917. 32983216 10.3389/fpls.2020.564917 PMC7485278

[34] Butelli E, Garcia-Lor A, Licciardello C, Casas GL, Hill L, Recupero GR, Keremane ML, Ramadugu C, Krueger R, Xu Q, et al. Changes in anthocyanin production during domestication of *citrus*. Plant Physiol. 2017;173(4):2225–42. 28196843 10.1104/pp.16.01701 PMC5373055

[35] Wang F, Lin J, Xu L, Peng Q, Huang H, Tong L, Lu Q, Wang C, Yang L. On higher nutritional and medical properties of a carotenoid-rich mutant pomelo (*Citrus maxima* (L.) Osbeck). Ind Crops Prod. 2019;127:142–7.

[36] Zhao C, Wang F, Lian Y, Xiao H, Zheng J. Biosynthesis of *citrus* flavonoids and their health effects. Crit Rev Food Sci Nutr. 2020;60(4):566–83. 30580548 10.1080/10408398.2018.1544885

[37] Huang D, Tang Z, Fu J, Yuan Y, Deng X, Xu Q. CsMYB3 and CsRuby1 form an ‘Activator-and-Repressor’ loop for the regulation of anthocyanin biosynthesis in *citrus*. Plant Cell Physiol. 2020;61(2):318–30. 31642503 10.1093/pcp/pcz198

[38] Thilmony R, Dasgupta K, Shao M, Harris D, Hartman J, Harden LA, Chan R, Thomson JG. Tissue-specific expression of *Ruby* in Mexican lime (*C. Aurantifolia*) confers anthocyanin accumulation in fruit. Front Plant Sci. 2022;13:945738. 36003820 10.3389/fpls.2022.945738 PMC9393592

[39] Chen L, Hu B, Qin Y, Hu G, Zhao J. Advance of the negative regulation of anthocyanin biosynthesis by MYB transcription factors. Plant Physiol Biochem. 2019;136:178–87. 30685697 10.1016/j.plaphy.2019.01.024

[40] LaFountain AM, Yuan YW. Repressors of anthocyanin biosynthesis. New Phytol. 2021;231(3):933–49. 33864686 10.1111/nph.17397 PMC8764531

[41] Xu WJ, Dubos C, Lepiniec L. Transcriptional control of flavonoid biosynthesis by MYB-bHLH-WDR complexes. Trends Plant Sci. 2015;20(3):176–85. 25577424 10.1016/j.tplants.2014.12.001

[42] Wang JH, Liu JJ, Chen KL, Li HW, He J, Guan B, He L. Anthocyanin biosynthesis regulation in the fruit of *Citrus sinensis* cv. Tarocco. Plant Mol Biology Report. 2016;34(6):1043–55.

[43] Rao MJ, Zuo H, Xu Q. Genomic insights into *citrus* domestication and its important agronomic traits. Plant Commun. 2021;2(1):100138. 33511347 10.1016/j.xplc.2020.100138 PMC7816076

[44] Liu Y, Zhang X, Liu X, Zheng P, Su L, Wang G, Wang X, Li Y, You C, An J. Phytochrome interacting factor MdPIF7 modulates anthocyanin biosynthesis and hypocotyl growth in apple. Plant Physiol. 2022;188(4):2342–63. 34983053 10.1093/plphys/kiab605 PMC8968312

[45] He ZH, Wang ZB, Nie XG, Qu M, Zhao HM, Ji XY, Wang YC. UNFERTILIZED EMBRYO SAC 12 phosphorylation plays a crucial role in conferring salt tolerance. Plant Physiol. 2022;188(2):1385–401. 34904673 10.1093/plphys/kiab549 PMC8825338

[46] Rubin G, Tohge T, Matsuda F, Saito K, Scheible W-R. Members of the LBD family of transcription factors repress anthocyanin synthesis and affect additional nitrogen responses in *Arabidopsis*. Plant Cell. 2009;21(11):3567–84. 19933203 10.1105/tpc.109.067041 PMC2798321

[47] Jiao P, Jiang Z, Wei X, Liu S, Qu J, Guan S, Ma Y. Overexpression of the homeobox-leucine zipper protein ATHB-6 improves the drought tolerance of maize (*Zea mays* L). Plant Sci. 2022;316:111159. 35151445 10.1016/j.plantsci.2021.111159

[48] Liu H, Shu Q, Lin-Wang K, Espley RV, Allan AC, Pei M, Li X, Su J, Wu J. DNA methylation reprogramming provides insights into light-induced anthocyanin biosynthesis in red pear. Plant Sci. 2023;326:111499. 36265764 10.1016/j.plantsci.2022.111499

[49] Telias A, Lin-Wang K, Stevenson DE, Cooney JM, Hellens RP, Allan AC, Hoover EE, Bradeen JM. Apple skin patterning is associated with differential expression of *MYB10*. BMC Plant Biol. 2011;11:93. 21599973 10.1186/1471-2229-11-93 PMC3127826

[50] Jiang S, Wang N, Chen M, Zhang R, Sun Q, Xu H, Zhang Z, Wang Y, Sui X, Wang S, et al. Methylation of MdMYB1 locus mediated by RdDM pathway regulates anthocyanin biosynthesis in apple. Plant Biotechnol J. 2020;18(8):1736–48. 31930634 10.1111/pbi.13337 PMC7336386

[51] Huang D, Wang X, Tang ZZ, Yuan Y, Xu YT, He JX, Jiang XL, Peng SA, Li L, Butelli E, et al. Subfunctionalization of the Ruby2-Ruby1 gene cluster during the domestication of *citrus*. Nat Plants. 2018;4(11):930–41. 30374094 10.1038/s41477-018-0287-6

[52] Sicilia A, Catara V, Scialò E, Lo Piero AR. Fungal infection induces anthocyanin biosynthesis and changes in DNA methylation configuration of blood orange *Citrus sinensis* L. (Osbeck). Plants-Basel. 2021;10(2):244. 33513740 10.3390/plants10020244 PMC7910907

[53] Liu HN, Shu Q, Kui LW, Espley RV, Allan AC, Pei MS, Li XL, Su J, Wu J. DNA methylation reprogramming provides insights into light-induced anthocyanin biosynthesis in red pear. Plant Sci. 2023;326:111499. 36265764 10.1016/j.plantsci.2022.111499

[54] Zhu YC, Zhang B, Allan AC, Kui LW, Yun Z, Ke W, Chen KS, Xu CJ. DNA demethylation is involved in the regulation of temperature-dependent anthocyanin accumulation in peach. Plant J. 2020;102(5):965–76. 31923329 10.1111/tpj.14680

[55] Martínez-Rivas FJ, Blanco-Portales R, Molina-Hidalgo FJ, Caballero JL, de Souza LP, Alseekh S, Fernie AR, Muñoz-Blanco J, Rodríguez-Franco A. Azacytidine arrests ripening in cultivated strawberry (*Fragaria* x *ananassa*) by repressing key genes and altering hormone contents. BMC Plant Biol. 2022;22(1):278. 35672704 10.1186/s12870-022-03670-1 PMC9172142

[56] Xu JD, Wang X, Cao HB, Xu HD, Xu Q, Deng XX. Dynamic changes in methylome and transcriptome patterns in response to methyltransferase inhibitor 5-azacytidine treatment in *citrus*. DNA Res. 2017;24(5):509–22. 28575160 10.1093/dnares/dsx021 PMC5737679

