# Supplementary material for: Identification of key genes controlling anthocyanin biosynthesis in the fruits of a bud variety of Tarocco blood-orange
Source: BMC Plant Biol. 2025 Feb 20;25:230. doi: 10.1186/s12870-025-06212-7 (PMC11841362; doi:10.1186/s12870-025-06212-7)
Supplement: Supplementary file 1 — Supplementary Material 1: Figure S1-S12 [file 12870_2025_6212_MOESM1_ESM.docx]

Fig. S1. Differential performance of the traits in Tarocco (WT) and its bud variant (MT). A, a bud-variant branch was found in a Tarocco orchard in Zeya, Wenzhou, China (120.41E, 28.02N), and showed significant suppression of fruit coloration compared with WT. B, after 30 d of post-harvest storage (4 °C), MT pulp was inhibited in the accumulation of anthocyanin compared with WT. C, at fruit maturity (235 DAF), the MT peel and pulp showed suppressed anthocyanin accumulation when compared to WT. D, MT leaf margins were crumpled when compared with the WT.

Fig. S2. The content of soluble sugars (A, including glucose, fructose, and sucrose) and organic acids (B, including citric, malic, and quinic acids) in the pulp of Tarocco (WT) and its bud variety (MT) at different development stages. Vitamin C (C) and carotenoid contents (D) in pulp at 280 days after flowering (DAF) in WT and MT varieties. Data are the means ± standard error (n = 3 biologically independent replicates). Asterisks indicate significant differences: ^∗^ *P* < 0.05.


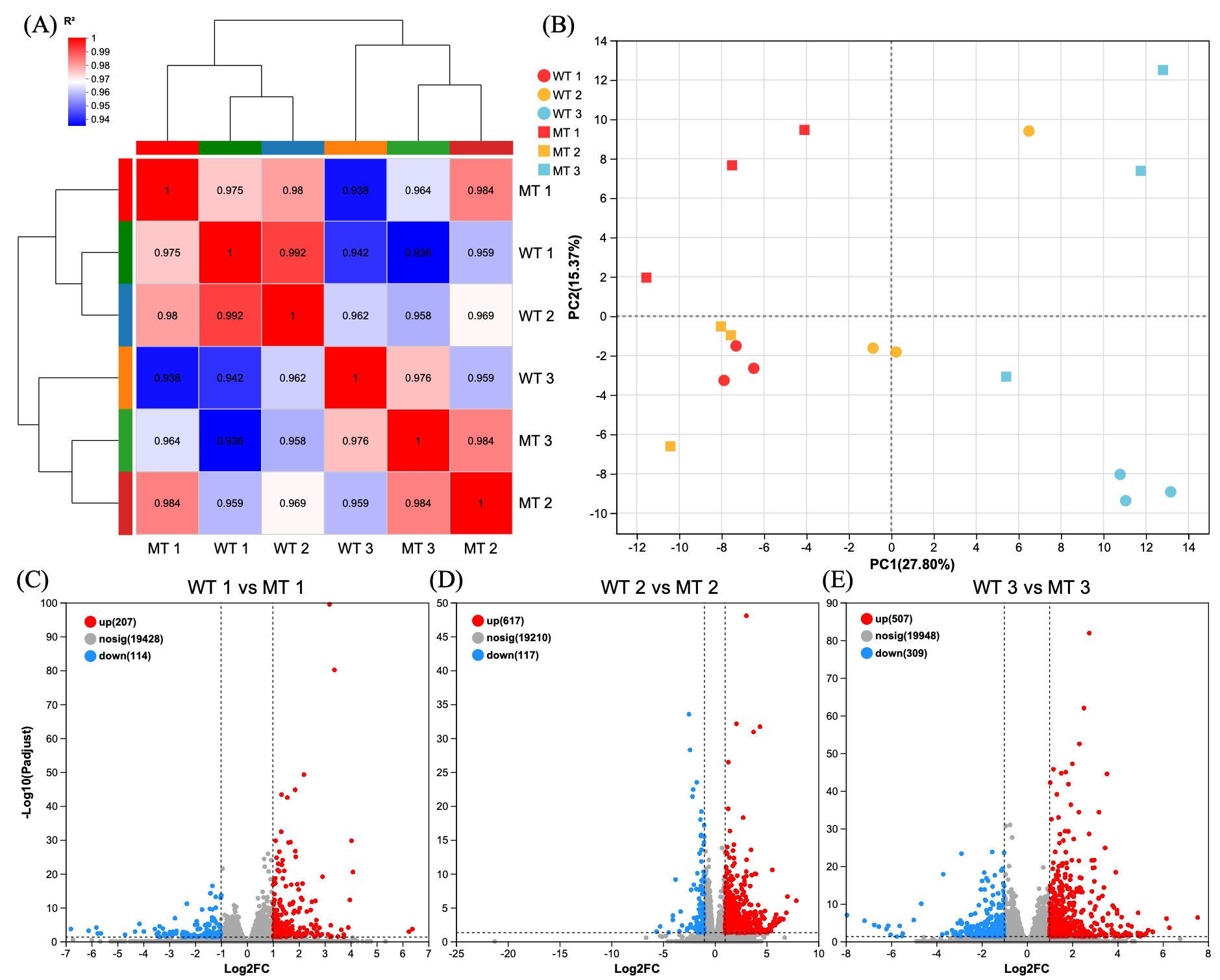


Fig. S3. Relationships between RNA-seq samples of Tarocco (WT) and their shoot variants (MT) at different developmental stages. A, inter-sample correlation. B, principal component analysis (PCA). C to E, differently expressed genes (DEGs). The pulp of WT and MT fruits collected at 235, 265, and 280 DAF was named WT1/MT1, WT2/MT2, and WT3/MT3, respectively.


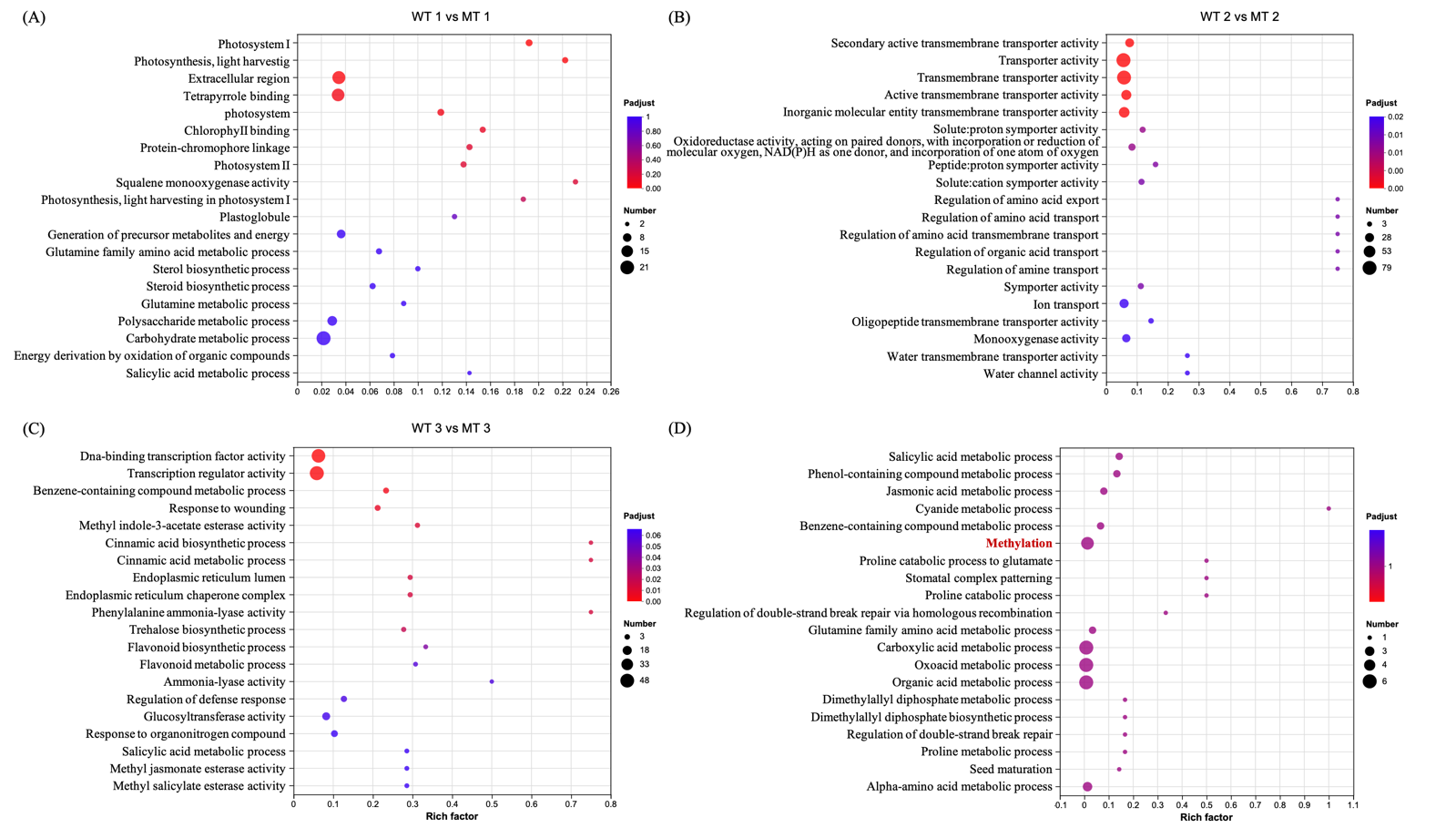


Fig. S4. GO enrichment analysis of the DEGs between WT1 vs. MT1 (A), WT2 vs. MT2 (B), and WT3 vs. MT3 (C), GO enrichment analysis of 64 DEGs common to the three compared groups (D). The pulp of WT and MT fruits collected at 235, 265, and 280 DAF was named WT1/MT1, WT2/MT2, and WT3/MT3, respectively.

Fig. S5. Cluster dendrogram of 9571 genes (A), 7 modules relationships of 9571 genes (B), correlation between the 7 modules (C and D).

Fig. S6. Transcriptome analysis to explain the differences in anthocyanin biosynthesis pathway genes and metabolites in the pulp of Tarocco (WT) and its bud variety (MT) at different stages of development. The heatmap displays the expression levels of the genes (average of TPM value) and metabolite [average of log10 (area value)], from left to right are WT 1, WT 2, WT 3, MT 1, MT 2, and MT 3. The panel shown in the upper left corner respectively indicate the amount of transcriptome (blue to red) and metabolite (green to red). The pulp of WT and MT fruits collected at 235, 265, and 280 DAF was named WT1/MT1, WT2/MT2, and WT3/MT3, respectively.

Fig. S7. qPCR validations of RNA-seq results. The histogram and the left-hand axis represent the results of qPCR, and the line graph and the right-hand axis represent the results of RNA-seq. The pulp of fruits collected at 235, 265, and 280 DAF was named stage 1, stage 2, and stage 3, respectively.

Fig. S8. Analysis of differences in the CDS of CsRuby from MT, WT, Navel (JN402329), Moro (JN402330.1), and Jingxian (JN402333) (A), the CsRuby CDS cloned from MT and overexpressed in MT pulp (B), and a comparison of the differences in total anthocyanin content (C), and expression levels of CsRuby (D) and anthocyanin synthesis genes (E) in the overexpression group (35S:CsRuby) and the control (35S) after transient expression.

Fig. S9. KEGG (A, B) and GO (C, D) enrichment analysis for up- and down-regulated DEGs of CK versus 5-aza, respectively.

Fig. S10. Relative proportions of CG, CHG, and CHH at CK and 5-aza (A). Hyper- and hypo-differentially methylated regions (DMRs) in CG, CHG, and CHH context in three comparative groups in circos plots (B-D).

Fig. S11. KEGG pathway enrichment analysis of DMRs in CG, CHG, and CHH context.

Fig. S12. Differentially methylated cytosines (DMCs) in CG, CHG, and CHH context of promoter and genebody of CsRuby (Cs_ont_6g005110) as viewed by IGV.
